# Transport of Thyroid Hormone in Brain

**DOI:** 10.3389/fendo.2014.00098

**Published:** 2014-06-24

**Authors:** Eva K. Wirth, Ulrich Schweizer, Josef Köhrle

**Affiliations:** ^1^Institut für Experimentelle Endokrinologie, Charité-Universitätsmedizin Berlin, Berlin, Germany; ^2^Institut für Biochemie und Molekularbiologie, Rheinische Friedrich-Wilhelms-Universität Bonn, Bonn, Germany

**Keywords:** blood–brain-barrier, transthyretin, deiodinase, flavonoids, endocrine disruptors, Mct8, l-type amino acid transporter, organic anion transporters

## Abstract

Thyroid hormone (TH) transport into the brain is not only pivotal for development and differentiation, but also for maintenance and regulation of adult central nervous system (CNS) function. In this review, we highlight some key factors and structures regulating TH uptake and distribution. Serum TH binding proteins play a major role for the availability of TH since only free hormone concentrations may dictate cellular uptake. One of these proteins, transthyretin is also present in the cerebrospinal fluid (CSF) after being secreted by the choroid plexus. Entry routes into the brain like the blood–brain-barrier (BBB) and the blood–CSF-barrier will be explicated regarding fetal and adult status. Recently identified TH transmembrane transporters (THTT) like monocarboxylate transporter 8 (Mct8) play a major role in uptake of TH across the BBB but as well in transport between cells like astrocytes and neurons within the brain. Species differences in transporter expression will be presented and interference of TH transport by endogenous and exogenous compounds including endocrine disruptors and drugs will be discussed.

## Molecules Involved in TH Transport in the Brain

The hydrophobic but amphipathic charged amino acid-derived thyroid hormones (TH) are carried and distributed by several binding proteins from their site of production, storage, and secretion, the thyroid gland, to their target tissues including the brain. In human blood, four major proteins, thyroxine-binding globulin (TBG), transthyretin (TTR), albumin, and apolipoprotein B 100 (ApoB100), bind more than 99% of the circulating TH T_4_, T_3_, and 3-iodo-thyronamine (3-T1AM). In contrast, only TTR has been found as one of the main proteins in CSF where it is produced and directionally secreted by choroid plexus (CP) epithelial cells into the liquor, which does not contain the high affinity TBG or the high capacity albumin TH binding proteins. Whether 3-T1AM, a TH-derived biogenic amine and its high affinity binding protein ApoB100 occur in CSF, remains to be studied.

Thyroid hormone enters the brain either directly via the blood–brain barrier (BBB) or indirectly via the blood–CSF-barrier (B–CSF-B), with the BBB route as the major entry path for the prohormone T_4_. T_4_ is locally metabolized by selenoenzymes to either active T_3_ via Type 2 deiodinase (Dio2) or inactivated by Type 3 deiodinase (Dio3), to yield reverse T_3_ (rT_3_). rT_3_, devoid of T_3_-like action, might be involved in developmental regulation of neuronal migration guided by astrocytes and glial cells (1). Dio2 is mostly expressed in astrocytes and tanycytes while Dio3 is mainly found in neurons. Whether Type 1 deiodinase (Dio1), catalyzing both 5′-deiodination (activation of T_4_ to T_3_) and 5-deiodination (inactivation of T_4_ and T_3_) is species-dependently expressed in brain remains controversial (2, 3). Dual entry paths of TH and cell type-specific expression of functional Dio enzymes in the brain raise the issues of (i) coordinated transport of active TH and TH metabolites between various brain cell types, (ii) organized communication between peripheral, thyroid-derived, and brain TH, and (iii) demands, supply, and disposal of TH precursors, metabolites, and active TH.

Adequate TH supply for the brain is of eminent importance during development but not less relevant in the differentiated adult organism with its changing hormonal requirement for metabolic and environmental adaptation.

Components controlling TH availability and action have been described in brain stem and progenitor cells (4) and TH receptor (TR) expression in the human brain has been demonstrated decades ago (5). Already during the first trimester human brain expresses various TH transporters (see Table 1), Dio enzymes, TR-isoforms, and isotypes in a development- and cell type-specific manner. Later in human pregnancy during weeks 17–20, endothelial cells and astrocytes organize the BBB [see Ref. (6)]. Endothelial cells express the TH transporter organic anion transporter polypeptide 1C1 (OATP1C1), which limits brain access of TH, especially T_4_. At this time point fetal thyroid already starts producing TH, thus disconnecting the fetal TH responsive system from the maternal source of TH, but still depending on further adequate maternal iodide supply.

**Table 1:** **Summary of expression profiles of thyroid hormone transmembrane transporters (THTT) in various cell types of the brain of several species**.

Transporter	Species	Areas of the brain	References
Slc16a2 (Mct8)	Mouse	Protein: cortex, hippocampus, cerebellum, choroid plexus, hypothalamus, tanycytes, vessels	(7–9)
	Human	Protein: cortex, hippocampus, choroid plexus, hypothalamus, tanycytes	
		Widespread expression in fetal brain	(6–8, 10–12)
	Rat	Protein: hippocampus, tanycytes, vessels	(8, 11, 13)
	Chicken	Transcript: brain	(14, 15)
	Siberian hamster	Transcript: hypothalamus	(16)
	Rabbit	Transcript: hypothalamus	(17)
	Zebrafish	Transcript: brain	(18, 19)
	Fathead minnow	Transcript: cortex, cerebellum, hypothalamus	(20)
	*Xenopus tropicalis*	Transcript: brain	(21)
Slc16a10 (Mct10)	Mouse	Transcript: cortex, hippocampus, choroid plexus	(7, 9)
	Human	Protein: cortex, choroid plexus, hypothalamus	(7, 10, 22)
	Rabbit	Transcript: hypothalamus	(17)
	Fathead minnow	Transcript: cortex, cerebellum, hypothalamus	(20)
	*Xenopus tropicalis*	Transcript: brain	(21)
Slc7a5 (Lat1)	Mouse	Transcript: hippocampus, choroid plexus	(7, 9)
		Protein: cortex, cerebellum	
	Human	Transcript: cortex	(7, 12)
	*Xenopus tropicalis*	Transcript: brain	(21)
Slc7a8 (Lat2)	Mouse	Protein: cortex, hippocampus, cerebellum, choroid plexus	(7, 9)
	Human	Protein: adult: cortex, hippocampus, choroid plexus; fetal: microglia	(7, 12)
Slco1c1 (Oatp14)	Mouse	Transcript: cortex, hippocampus	(7–9, 23)
		Protein: choroid plexus, tanycytes, vessels	
	Human	Transcript: cortex	(7, 8, 10, 12, 22)
		Protein: choroid plexus, hypothalamus	
	Rat	Protein: choroid plexus, vessels	(8, 24)
	Chicken	Transcript: brain	(14, 15)
	Rabbit	Transcript: hypothalamus	(17)
	Fathead minnow	Transcript: cortex, cerebellum, hypothalamus	(20)
	*Xenopus tropicalis*	Transcript: brain	(21)

*If available, protein data is preferably mentioned. Transcript data is only mentioned if no or only minimal protein data is available*.

Facilitated uptake and release of TH by TH transmembrane transporters (THTT) is essential for their intracellular availability. TH have to cross multiple membranes in order to reach their nuclear and mitochondrial receptors. Especially, the entry of TH into the brain via the BBB and their subsequent distribution throughout all brain areas poses challenges in form of membranes of different cell types to be crossed. The complex interaction and communication between astrocytes and neurons, demonstrated for metabolic as well as synaptic processes, is in place regarding TH metabolism and distribution throughout the brain. Therefore, the specific spatio-temporal distribution of TH in different areas and cell types of the brain is required during embryonic development and differentiation, but also for adult maintenance and regulation of brain activity and metabolism.

## Role of TH Binding and Distributor Proteins for TH Availability to Brain Cells

Tissue and cellular uptake depends on free TH concentrations in blood and both free T_4_ and free T_3_ are available for cellular uptake by THTT, while TH bound with high affinity to serum distributor proteins TBG and TTR is assumed not to be directly available for cellular uptake during organ perfusion by blood (25). In contrast, TH bound to albumin with high capacity but low affinity is easily liberated based on the high TH off-rate constants shown in liver perfusion (26, 27). While peripheral sensory neurons internalize TTR in megalin-dependent manner in context of neuritogenesis *in vitro* (28), evidence is missing, that TH–TTR ligand–protein complexes are taken up by neurons, astrocytes, or glial cells via receptors for these proteins expressed on brain cells. Such “trojan horse” mechanisms of ligand transmembrane transfer have been demonstrated during fetal development for several protein bound (pro-)hormones and vitamins such as TH, retinol, vitamin D3, and steroid hormones (29–31) for several peripheral target tissues including adult kidney but not yet for the fetal brain. Such mechanisms, mediated by megalin or cubilin receptors, might provide entry routes for hormones bypassing the concept of the “free hormone hypothesis” (32) and use of THTT.

Independent from such a mechanism, the high expression of evolutionary conserved TTR in CP and meninges of the developing and adult brain offers a further exchange compartment for TH, especially T_4_, a high affinity ligand of TTR. During fetal brain development, the prominent voluminous CP is required for proper CNS growth and differentiation. TTR is the only TH binding protein expressed and directionally secreted into CSF. TTR, first described in CSF and subsequently in plasma, constitutes up to 20% of CSF protein and its secretion into CSF starts already in fetal week 8 before its hepatic production (28). Whether its adequate function as binding and distribution protein for two major morphogen precursors, T_4_ and RBP-bound retinol, is essential or redundant, remains to be established. Lack of a major phenotype of TTR gene inactivation in the mouse came as a surprise with respect to normal brain development, HPT axis, TH homeostasis, and retinol-dependent functions (33). These mice grew normal, were fertile and had normal tissue T_4_ levels though plasma TH concentrations were significantly reduced. Apparently, lack of TTR can be compensated by a shift of TH binding to rodent TBG, which has lower TH affinity compared to human TBG (34). TH metabolism and T_3_-responsive gene expression of HPT axis and liver was unchanged in TTR knock-out mice. These observations either suggest a minor role for TTR-dependent TH binding and directional transport into CSF for proper brain development (35) or indicate existence of still unknown compensatory mechanisms active in absence of TTR during mouse development. At least this mouse model did not support the hypothesis that hormone binding and distribution proteins such as TTR in case of TH directly contribute to cellular hormone uptake as proposed by Pardridge (36) in distinction to several observations by the group of Willnow (31).

In contrast to these observations in the TTR knock-out mouse model are some findings on effects of endogenous or exogenous ligands of TTR, such as (iso-)flavonoids and endocrine disruptors, interfering with TH homeostasis in circulation, in CNS, and during fetal development. Several natural flavonoids, secondary metabolites of plants contained in our regular diet, avidly bind to TTR and displace TH from TTR binding based on their structural resemblance to TH. Resulting elevated free TH blood concentrations increase renal TH loss (transiently), elevate TH tissues levels, and enhance TH transfer via the placenta into fetal circulation including the fetal brain. *In vitro* as well as rodent animal studies provided evidence for interference of flavonoids and other TTR binding endocrine disruptors with CP-derived TTR-mediated TH transfer into CSF and the brain, resulting in disturbed homeostasis of brain TH levels and bioavailability (37).

More studies are needed analyzing (i) interference by natural and synthetic flavonoids with TH transport, (ii) action of endocrine disrupters such as the flame retardants polybrominated diphenylethers (PBDE) during neuronal stem cell development (38), or (iii) impact of ligands and pharmaceuticals, structurally related to TH (39) and interfering with THTT function (40). Recently, molecular actions and biological functions have been initially characterized for so far “neglected or minor” endogenous TH metabolites such as acetic acid- (Tetrac, Triac) or amine-derivatives (3-T1AM) of TH (41) and 3,5-T2, the latter abundantly present in the CNS (42). This raises the questions, (i) whether they are active players in TH-regulated brain function during development and in the adult organism, (ii) whether and how these metabolites are generated and transported in the brain, and (iii) how their mode of action interferes with classical TH action, which is mainly mediated via T_3_-liganded TR. New modes of action may be envisaged for these TH metabolites at the plasma membrane, on cytosolic signaling cascades, or on other subcellular compartments of brain cells (43–45).

## THTT among Species

Many transporters have been shown to transport TH. The most specific THTT is the monocarboxylate transporter 8 (Mct8; Slc16a2). Up to date, it is the only transporter with TH as the exclusive substrate. All other transporters out of the classes of monocarboxylate transporters (MCT), organic anion transporting polypeptides (OATP), and l-type amino acid transporters (LAT) also transport other substrates like amino acids. Most data about the presence and localization of THTT has been generated in mice and humans. The following table summarizes expression of the most researched THTT Mct8, Mct10, Lat1, Lat2, and Oatp1c1 in various vertebrate species (Table 1).

Research focus has been on the only THTT identified to cause a human disease so far, i.e., Mct8. MCT8 is widely expressed in the human fetal brain in several cell types (Table 1). Strong transcript and protein signals were observed in the cortical plate and subplate, as well as in ventricular and subventricular zones. Throughout fetal development CP epithelial cells and ependymal cells express high levels of MCT8 (6–8).

Comparably high MCT8 expression has been reported for monkey brain, which expresses OATP1C1 and LAT1 albeit at much lower levels (46). MCT8 mutations cause a severe syndrome of psychomotor retardation, the Allan–Herndon–Dudley syndrome (AHDS) (47–50). This syndrome also comprises endocrine manifestations with high circulating T_3_, low T_4_, and normal to elevated TSH levels. The mouse model for *Mct8*-deficiency replicates the endocrine phenotype, but it does not mimic the psychomotor retardation of the human syndrome (7, 51, 52). Since AHDS is not comparable to the classical phenotype of congenital hypothyroidism or cretinism, it is of great importance to identify THTT in brain areas of human brains, as well as in the model organism used for analyzing TH transport to be able to understand phenotypic variations between these models. Other animal models apart from *Mct8*-deficient mice will be needed to evaluate the involvement of THTT in basic brain development. Recently, zebrafish has been evaluated for developmental effects of *Mct8*-deficiency. Significant species differences with respect to cell types, time point, dynamics, and regulation of THTT especially in the developing but also the adult brain have been reported for humans, monkey, chicken, rodent, fish, and amphibian brain (Table 1). For example, we demonstrated the expression of an additional transporter, Lat2, in mouse neurons during development, which is not expressed in human developing neurons (7). Research on double knock-out mice of Mct8 with either Mct10 or Oatp1c1 yielded valuable data on the interplay and possible compensation between these transporters (53, 54). Simultaneous deletion of *Mct8* and *Oatp1c1* lead to the ablation of both T_3_ and T_4_ transport across BBB. Symptoms of brain hypothyroidism were intensified underlining the importance of THTT for proper brain development and function. Further research analyzing the developmental expression of THTT and comparing effects of loss of function among different species will yield important information on the temporal effects of these transporters on brain development.

## Structural Aspects of THTT

Thyroid hormone transport proteins MCT8, MCT10, OATP1C1, as well as LAT1 and LAT2 belong to different subfamilies within a huge protein superfamily of transport proteins, the major facilitator superfamily, MFS (55). General insights into the function of such transmembrane proteins can be derived from pathogenic mutations, e.g., in MCT8 from patients affected by AHDS (56).

Substrate recognition by transport proteins is fundamentally different from ligand binding in, e.g., nuclear receptors: while receptors are optimized for high affinity binding of their ligands, this would be detrimental for transporters, as these have to release their substrates easily. Most receptors have one binding site, while THTT should have at least two – one accessible from the exterior and one accessible form the interior of the cell. These two binding sites may overlap and differ only according to conformational changes associated with transport. The question is thus, how can a transporter achieve specificity and at the same time prevent tight binding?

With the exception of MCT8, all other THTT transport additional substrates, namely amino acids, bile acids, or conjugated steroids. It should therefore be of particular interest to compare how different protein families have adapted to transport TH and whether the substrate–protein interactions are similar or not. Experimental structures are not available for any of the THTT. Homology models based on experimental structures have been created for OATP1C1 (57), MCT8 (39), and LAT1 (58).

The homology model of rat Oatp1c1 was based on three high resolution crystal structures of bacterial transport proteins, lactose permease LacY, glycerol–phosphate transporter GlpT, and multidrug resistance protein EmrD. Authors achieved similar models with all templates and highlighted sequence identities between Oatp1c1 and templates below 10%. Conserved amino acids between Oatp1c1 and bacterial transporters were known to be functionally important in LacY and GlpT and may therefore not be involved in substrate specificity. While this work nicely shows that Oatp1c1 conforms to the overall structure of the bacterial transporters, there is no information on how specificity is established (57).

Chemical probes reactive with cysteines (*p*-chloromercury benzenesulfonate, pCMBS) or histidines (diethylpyrocarbonate, DEPC) were used to modify MCT8 and to test its activity afterward (59, 60). This approach suggested that Cys481, Cys497, and His192 may be close to the substrate translocation channel. Mutation to Ala of these critical amino acids rendered MCT8 resistant to pCMBS and DEPC.

We created a MCT8 homology model based on the inward-open conformation of GlpT and identified two charged amino acids within the transmembrane domains, Asp498 and Arg445, which are essential for transport (39). The homology model predicted a salt bridge between both residues. We suggested interaction of TH carboxyl and amino groups with these amino acids during transport, since TH analogs lacking the carboxyl or amino groups are not transported by MCT8 (39). The salt bridge was later independently confirmed by charge reversal mutants (61). MCT8 accepts only L-T_3_, L-T_4_, L-rT_3_, and L-3,3′-T_2_ as substrates (39). Based on the occurrence of a His–Arg clamp pinching T_3_ in the T3 receptor β structure, we tested the hypothesis that a His–Arg pair spaced by about 15Å could serve the same purpose in MCT8 (62). Mutation of His192 (which may work together with Arg445 in an outward open conformation) clearly demonstrated His192 participation in substrate recognition (63). Interestingly, His192 corresponds to Gln88 in MCT10, a closely related homolog of MCT8 unable to transport T_4_ (64). Mutation of His415 and Arg301, conserved in MCT8 and MCT10, affected transport kinetics as expected from substrate interactions (63). These findings corroborate the usefulness of the MCT8 homology model and suggest how the substrate is bound by MCT8 – at least in the inward-open conformation.

Recently, a LAT1 homology model was presented based on the crystal structure of bacterial agmatine antiporter AdiC (58). Iodotyrosines were identified *in silico* as LAT1 substrates and confirmed experimentally. Interestingly, while carboxy and amino groups are present in all LAT1 substrates, modeling suggests that these functions are sampled by the transporter by backbone polar contacts instead of side chain contacts as predicted in MCT8 (58, 63). Different transporters may have adapted different strategies to recognize iodinated TH substrates.

## THTT in Cell Types of the Brain

Analyses of brain regions provide important insight into THTT distribution. Immunohistochemical staining for Mct8 in mouse brains did not only show typical neuronal staining patterns, but also staining in astrocytes, CP, and tanycytes (7). However, most techniques like *in situ* hybridization or immunohistochemistry do not allow for a real cell type-specific resolution and detection. Effects of TH uptake into neurons vs. astrocytes can not be dissected in complete brains. Primary cultures of mouse brain cells facilitate selection of a single cell type and the evaluation of effects of THTT deletion. Primary cultures of mouse neurons, astrocytes, and microglia can be used to detect THTT expression and functionality, as well as the reaction of specific cells on different conditions of TH access. Genetically engineered mouse lines can be used to create cell cultures originating from wildtype and transporter-deficient mice. Employing these cultures, we detected expression of Mct8, Lat1, and Lat2 transcripts and proteins in neurons and astrocytes, while Lat2 is additionally expressed in microglia (9, 65). Functional uptake studies in *Mct8*- and *Lat2*-deficient primary neuronal and astrocyte cell cultures demonstrated involvement of both transporters in T_3_ and T_4_ uptake into neurons and astrocytes (7, 9). The fraction of TH uptake mediated by transporters of the Mct, Lat, and Oatp groups can also be monitored by utilizing inhibitors of these transporters. Transport by Mct can be inhibited with bromosulphophtalein (BSP), while 2-aminobicyclo-(2,2,1)-heptane-2-carboxylic acid (BCH) is a Lat inhibitor and probenicid inhibits transport by Oatps. Uptake studies with these inhibitors gave very similar results on the involvement of these groups of transporters in TH uptake to genetic inactivation (7, 9).

Up to date, all concepts of interaction and cooperation between neurons and astrocytes imply astrocytes as providers of energy, communication and T_3_ for neurons. However, it has been shown that neurons are generally able to carry out all functions needed for energy metabolism by themselves. It is therefore quite possible that neurons are able to convert T_4_ to T_3_ by expressing functional Dio2. Cell type-specific animal models and primary cell cultures are of great importance to study the interaction of neurons and astrocytes regarding transfer of TH metabolites and regulation of deiodinases independent of TH uptake at the BBB.

## Conflict of Interest Statement

The authors declare that the research was conducted in the absence of any commercial or financial relationships that could be construed as a potential conflict of interest.
